# Supplementary material for: A qualitative exploratory study of UK first-time fathers’ experiences, mental health and wellbeing needs during their transition to fatherhood
Source: BMJ Open. 2019 Sep 13;9(9):e030792. doi: 10.1136/bmjopen-2019-030792 (PMC6747673; doi:10.1136/bmjopen-2019-030792)
Supplement: Supplementary data [file bmjopen-2019-030792supp001.pdf]

Appendix – A

NEW DAD STUDY (NEST)

CONSENT FORM – Study Phase 2 for first time fathers

Rec Reference Number: 17/LO/0815

Title of Project: New Dad Study (NEST)

Name of Researcher taking consent:

Please initial box

1. I confirm that I have read the information sheet dated 05/06/17 (version -3) for the above study. I have had the opportunity to consider the information, ask questions and have had these answered satisfactorily. ☐
2. I understand that my participation is voluntary and that I am free to withdraw at any time without giving any reason, without my medical care or legal rights being affected. ☐
3. I understand that the interview will be audio- recorded. ☐
4. I understand that the information collected about me will be used to support other research in the future, and may be shared anonymously with other researchers. ☐
5. I agree to take part in the above study. ☐

____________________	____________________	____________________
Name of Participant	Date	Signature

____________________	____________________	____________________
Name of Person taking consent	Date	Signature

When completed: 1 for participant; 1 for researcher site file.

New Dad Study (NEST) Version - 3, 05/06/17 IRAS Study Number: 203629
