# Supplementary material for: A qualitative exploratory study of UK first-time fathers’ experiences, mental health and wellbeing needs during their transition to fatherhood
Source: BMJ Open. 2019 Sep 13;9(9):e030792. doi: 10.1136/bmjopen-2019-030792 (PMC6747673; doi:10.1136/bmjopen-2019-030792)
Supplement: Supplementary data [file bmjopen-2019-030792supp002.pdf]

Appendix – B

NEW DAD STUDY (NEST)

Topic Guide for Qualitative Interviews

1. Tell me about your experience of becoming a father for the first time? How do you feel about it?

2. How prepared did you feel? What information helped you to prepare for becoming a father?

3. How has becoming a father impacted on your emotional wellbeing?

4. How have you coped with the changes of becoming a dad?

5. How would you describe your relationship with your partner? Have you noticed any changes? How have you been getting on since becoming a father?

6. What support systems do you have in place? What other support/resources have you accessed (*online resources or support)?*

7. How was the support from health professionals (*MW, HV, GP both antenatally and postnatally)?*

8. What helpful information, resources or advice did you received about becoming a father (*such as leaflets, websites, forums, antenatal/ postnatal classes, health promotion sessions, visit from health visitor)?*

9. What other information/ resources do you think would have been useful?

10. What have been the barriers to accessing help/ support?

11. What would enable you to better access help or support?

12. When would be the best time for receiving support or information about emotional wellbeing relating to becoming a father? *E.g. – start of pregnancy, later stages of pregnancy, soon after the baby is born, ongoing etc.*

This is the end of the interview. I would like to thank you very much for taking part in this study. Do you have any questions you would like to ask?

New Dad Study (NEST) Version - 1, 09/02/17 IRAS Study Number: 203629
